# Pharmacists’ role in multidisciplinary diagnosis and treatment in adverse reactions: A case report of interferon alfa-2b induced severe lupus

**DOI:** 10.1097/MD.0000000000031997

**Published:** 2022-12-16

**Authors:** Hongxia Chen, Xiaoyan Qiu, Jingyi Wang, Hualing Wei

**Affiliations:** a Department of pharmacy, Fudan University Huashan Hospital, Shanghai, China; b Department of clinical pharmacy, People’s Hospital of GuangXi Zhuang Autonomous Region, Nanning, Guangxi, China.

**Keywords:** adverse reaction, clinical pharmacist, interferon, multidisciplinary team, systemic lupus erythematosus

## Abstract

Various side effects of interferon alfa-2b (IFN-*α*2b) have been reported. However, no relevant research has been conducted on the identification and treatment scheme selection for IFN-*α*2b induced severe systemic lupus erythematosus (SLE).

**Patient concerns:**

A 41-years-old man with a long history of hepatitis B who developed severe active SLE after IFN-*α*2b therapy for 24 months, with complete and persistent remission of clinical and laboratory abnormalities after IFN-*α*2b withdrawal, was not observed.

**Diagnosis:**

The patient was diagnosed with interferon-associated lupus by a multidisciplinary team involving pharmacists, and lupus nephritis by renal biopsy.

**Interventions:**

Methylprednisolone (40 mg/day) with intravenous cyclophosphamide (600 mg/body weight) was initiated and the symptoms were partially relieved. Cyclophosphamide was increased from 600 mg to 850 mg at the pharmacist’s recommendation.

**Outcomes:**

The patient showed a favorable response to these therapies.

**Lessons:**

Clinical pharmacists collaborated with other members of the health care team to diagnose and treat adverse reactions, resulting in improved patient management.

## 1. Introduction

HBV (Hepatitis B virus) infection is a major global health problem that causes acute and chronic liver diseases that can lead to liver cirrhosis and hepatocellular carcinoma.^[1]^ Interferon alfa-2b (IFN-*α*2b) has been recommended by the World Gastroenterology Organization Global Guidelines as a first-line strategy for HBV treatment owing to its significantly improved outcomes. Hence, it is widely used in a large number of hepatitis B patients, particularly Chinese patients.^[2,3]^ With the widespread use of IFN-*α*2b, mild-to-moderate adverse drug reactions (ADR) occur, including eruptions,^[4,5]^ pustular psoriasis,^[6]^ Bell’s palsy,^[7]^ and hepatic granulomas.^[8]^

ADR may be defined as any noxious, unintended, or undesired effect of a drug, excluding therapeutic failures, intentional and accidental poisoning, and drug abuse.^[9]^ The presentation of ADR in adults is often atypical, which further complicates recognition. Accurate identification of serious ADR is extremely important as it provides evidence for suspected drug withdrawal. Once delayed, patients’ physical functions or organs may suffer serious or irreversible damage. Clinical pharmacists are uniquely trained to impact medication safety for individual patients through medication management skills that are part of the clinical pharmacist’s role as well as to analyze the performance of medication processes and design individual treatment strategies to mitigate drug-related outcomes that may cause harm.

Here, we report the case of a clinical pharmacist involved in the diagnosis and therapy of IFN-*α*2b induced ADR. As part of a multidisciplinary team, clinical pharmacists identified systemic lupus erythematosus (SLE) as an IFN-*α*2b related ADR and provided individual treatment regimens and dosages to physicians according to the patient’s age, liver function, risk of infection, renal injury indicators, extrarenal organ injury, fertility intention, comorbidities, and response to treatment with previous immunity inhibitors. Furthermore, this case provides insights into the clinical treatment of these patients.

## 2. Ethical approval

The patient gave written informed consent for the use of his medical data.

## 3. Case presentation

The patient was an early 40s Chinese male with a history of hepatitis B for several decades. There had no specific family history of smoking or alcohol abuse. He underwent surgery for hepatic angioleiomyoma in 2018 and received 10 million IU IFN-*α*2b thrice a week since 2019. Approximately 12-months after the IFN-*α*2b course, HBsAg loss and seroconversion appeared to be much better than those in the previous treatment, and IFN-*α*2b therapy was continued. In November 2021, at the end of 24-month of IFN-*α*2b therapy, his blood test results suggested thrombocytopenia (thrombocyte count,88 × 10^9^/L); however, the patient attempted to continue treatment. Unfortunately, pancytopenia (white blood cell count 3.78 × 10^9^/L; hemoglobin, 108 g/L; and thrombocyte count, 71 × 10^9^/L), hypoalbuminemia (albumin 25.4 g/L), and abnormal creatinine (105*μ*mol/L) with symptoms of mild pedal edema, proteinuria, and hematuria were noted when he finished the last dose of the second course. The patient was admitted to our department due to symptom deterioration.

### 3.1. Initial investigations

On examination, the temperature was 36.6ºC, heart rate was 78 beats/minute, blood pressure was 129/105 mm Hg, and oxygen saturation was 99%. heart sounds were normal and there were no signs of heart failure or tamponade. Eyelid swelling and bilateral leg pitting edema up to the knee were also observed. However, there was no hair loss, malar rash, or tenderness of the abdomen or joints. No palpable lymphadenopathy is observed.

**Figure 1.**
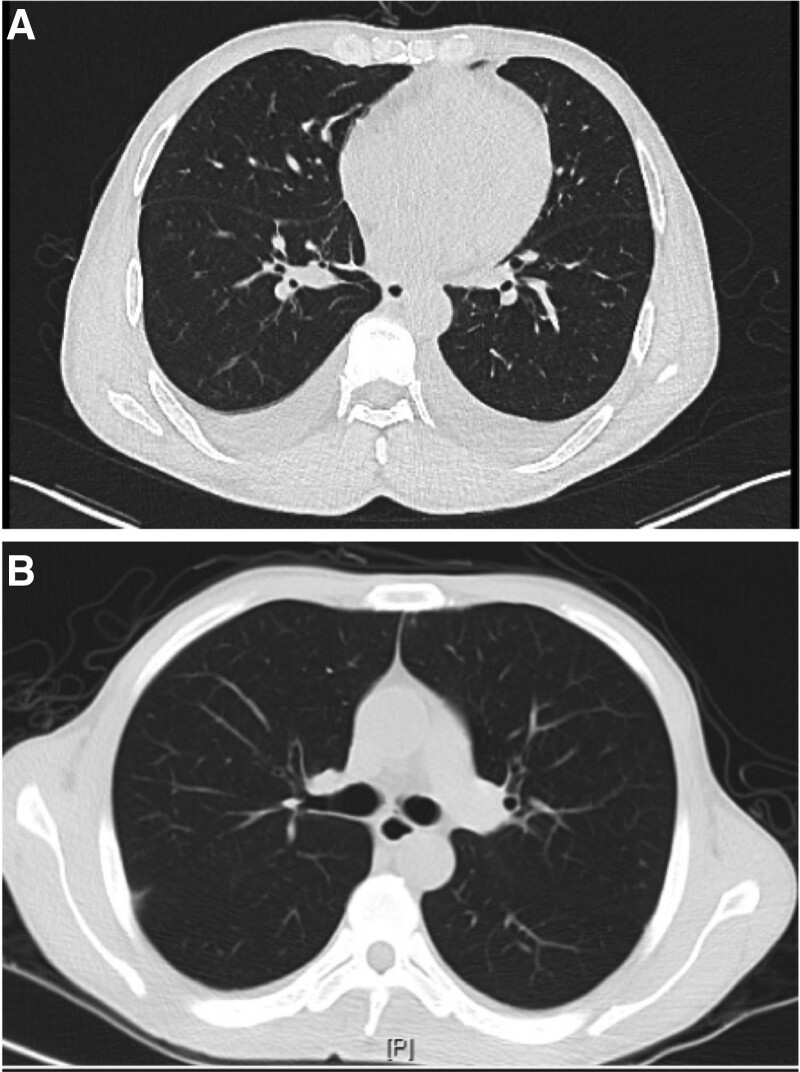
(A) Computerized tomography (CT) scan of further examination on admission, showing pleural effusion (arrowhead). (B) CT showed that the pleural effusion disappeared after treatment. CT = Computed tomography.

**Figure 2.**
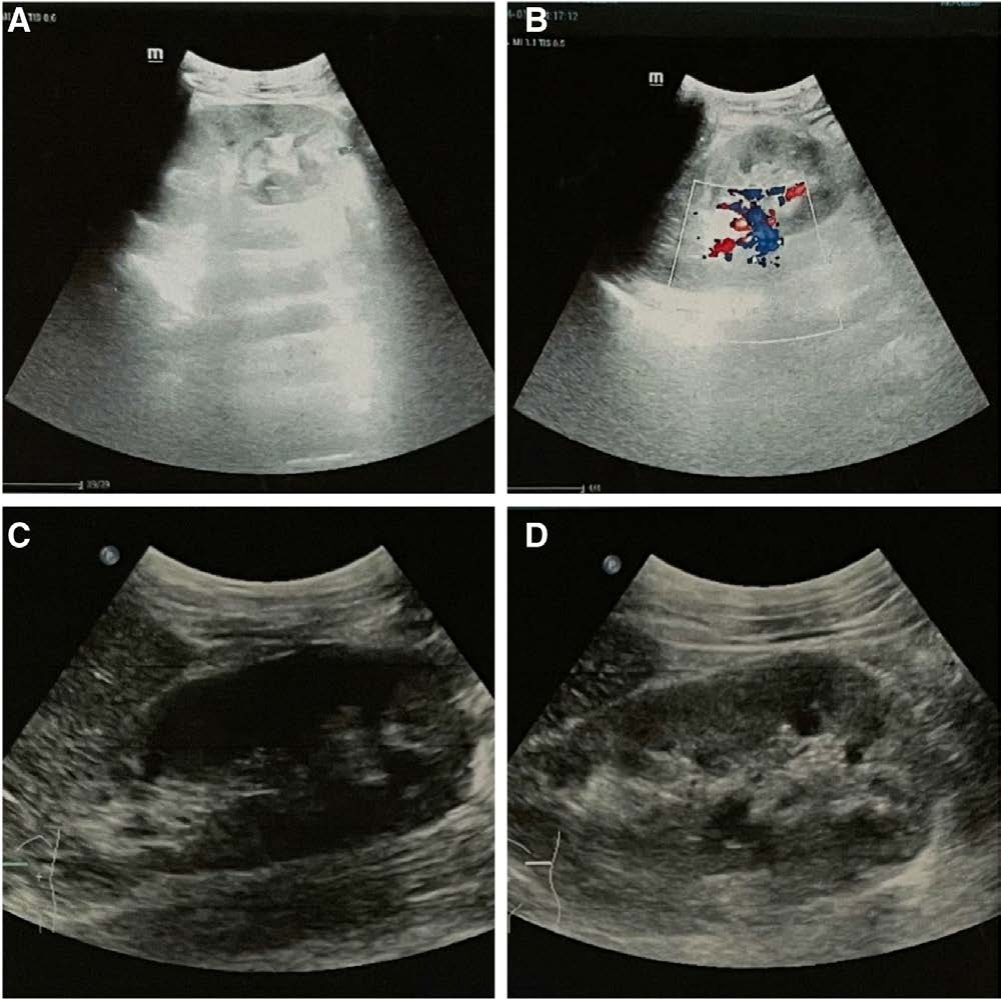
(A) (B) On admission, abdominal ultrasound showed the shape and size of both kidneys were normal, along with clear demarcation of renal cortex and medulla without hydronephrosis and abnormal echoes. (C) (D) Abdominal ultrasound showed no abnormalities in both kidneys after 6 courses of cyclophosphamide.

**Figure 3.**
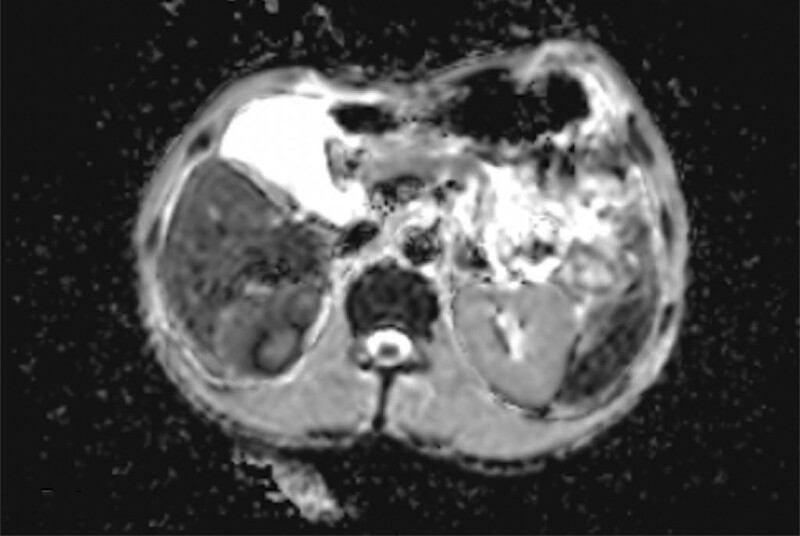
Further examination liver Nuclear Magnetic Resonance (MR) scan, showing a normal signal.

Laboratory results (Table 1) showed a total leukocyte count of 2.63 × 10^9^/L, hemoglobin count of 87 g/L, platelet count of 69 × 10^9^/L, and normal neutrophil, lymphocyte, neutrophil, and lymphocyte counts. Patients with iron, vitamin B12, or folic acid deficiencies were excluded. Immunofixation electrophoresis was normal and not suggestive of other haematological malignancies. Urine examination showed occult blood (+++), protein (++), cellular casts (+), urinary protein 8.58g/24 hours, microalbuminuria 9260.00 mg/L, and biochemical evaluation revealed a serum albumin level of 18 g/L and creatinine level of 120*μ*mol/L, which was worse than a month ago; however, renal biopsy could not be performed due to severe anemia and thrombocytopenia. myeloperoxidase and proteinase-antineutrophil cytoplasmic, anti-phospholipase A2 receptor (PLA2R), and anti-glomerular basement membrane antibodies were negative, ruling out the association of primary antineutrophil cytoplasmic antibodies-associated nephritis, membranous nephropathy, and anti-glomerular basement membrane antibody glomerulonephritis, respectively.^[10]^ The antinuclear antibody (ANA) (1:1000) and anti-double stranded DNA (dsDNA) antibody (581.0 IU/mL) tests were positive, and the hypocomplementemia and decreased Immunoglobulin G levels were significant, indicating that they might be closely associated with lupus erythematosus. Most liver indicators were within the normal range and the copy number of HBV DNA was < 50 IU/mL. Computed tomography of the chest confirmed the presence of fluid in the pleural cavity (Fig. 1A). Abdominal ultrasonography showed that the shape and size of both kidneys were normal, along with a clear demarcation of the renal cortex and medulla, without hydronephrosis or abnormal echoes (Fig. 2A, 2B). No abnormal signals were observed in the liver nuclear magnetic resonance (MR) scan (Fig. 3).

**Table 1:** Laboratory data on admission.

Blood cell count		Biochemical data		Antibody data	
White blood cells (×109/L)	2.63	Aspartate aminotransferase (U/L)	43	Anti-nuclear antibody	1:1000
Neutrophils (%)	66	Alanine aminotransferase(U/L)	35	Anti-dsDNA antibody (IU/mL)	581.0
Lymphocytes (%)	24	Total bilirubin (*μ*mol/L)	5.0	MPO-ANCA (RU/mL)	<2.0
Haemoglobin (g/L)	87	Direct bilirubin (*μ*mol/L)	1.4	PR3-ANCA (RU/mL)	2.3
Mean Corpuscular Volume (fl)	88.3	Alkaline phosphatase (U/L)	91	Anti-nRNP/Sm antibody	(-)
Haematocrit (%)	25.6	Creatine kinase (U/L)	122	Anti-Sm antibody	(-)
Platelets (×109/L)	69	Lactate dehydrogenase (U/L)	377	Anti-SSA antibody	(+)
		*α*-hydroxybutyrate dehydrogenase (U/L)	293	Anti-RO-52 antibody	(-)
Anemia data		Total protein (g/L)	36	Anti-SSB antibody	(-)
Erythropoietin (IU/L)	10.5	Albumin (g/L)	18	Anti-Scl-70 antibody	(-)
VitaminB12 (pg/mL332)	332.0	Globulin (g/L)	18	Anti-PM-SCL100 antibody	(-)
Folic acid (ng/mL)	4.90	Urea (mmol/L)	10.9	Anti-Jo-1 antibody	(-)
		Uric acid (mmol/L)	0.590	Anti-nucleosome antibody	(+)
Coagulation data		Creatinin (*μ*mol/L)	120	Antihistone antibody	(+)
International Normalized Ratio	0.96	eGFR (mL/min)	64.8	Anti-ribosomal P protein antibodies	(-)
Prothrombin time (s)	11.1	Potassium (mmol/L)	4.5	Anti-mitochondrial M2 antibody	(-)
Partial thromboplastin time (s)	23.7	Sodium (mmol/L)	147	Anticardiolipin antibody (RU/mL)	5.7
Fibrinogen (g/L)	3.7	Chloride (mmol/L)	117	Anti-glomerular basement membrane antibody (RU/mL)	<2.0
Thrombin time (s)	17.3	Calcium (mmol/L)	1.68	Anti-phospholipase A2 receptor antibody (RU/ml)	<2.0
D-dimer (FEUmg/L)	2.06	Phosphorus (mmol/L)	1.11	Anti-GBM antibody (RU/mL)	<2.0
		Magnesium (mmol/L)	0.92		
Urinalysis		Rheumatoid factor (IU/mL)	<10.60	HBV data	
Voided volume (L/24h)	2200	IgG(g/L)	6.55	HBSAg (IU/mL0.0)	0.00
Urinary protein (g/24 h)	8.58	IgA (g/L)	1.42	HBSAb (IU/L > 100)	> 1000.0
Blood	(+++)	IgM (g/L)	1.03	HBeAg (s/co)	0.28 (-)
Urine cast	(+)	IgG4 (g/L)	0.130	HBeAb (s/co)	0.0
White blood cells (/*μ*L)	122.4	C3 (g/L)	0.241	HBcAb (s/co)	8.3
urine creatinine (mmol/L)	16.51	C4 (g/L)	<0.068	HBV-DNA (IU/mL)	<50
Microalbuminuria (mg/L)	9260.00				

ANCA = antineutrophil cytoplasmic antibodies, dsDNA = double stranded DNA, GBM = glomerular basement membrane, IgG = Immunoglobulin G, MPO = myeloperoxidase, PR3-ANCA = proteinase-antineutrophil cytoplasmic.

The SLE Disease Activity Index score was 24 points (Table 2), and the British Isles Lupus Assessment Group Index (BILAG) score was 19 points:1A (nephritis), 3B item (leukopenia, erythrocytopenia, thrombocytopenia) and 1C item (pleural effusion). The diagnosis of lupus nephritis (LN) was based on proteinuria (++), abnormal protein-to-creatinine ratio (4958.12 mg/g), and positive urine sedimentation (malformed erythrocytes, erythroid, and leukocyte-type tubes) in lupus diagnosis.^[10]^

**Table 2:** The Patient’s SLE disease activity index (SLEDAI) score.

No	SLEDAI item	Description	Weight
1	Seizure	Recent onset. Exclude metabolic, infectious or drug cause.	0
2	Psychosis	Altered ability to function in normal activity due to severe disturbance in the perception of reality. Include hallucinations, incoherence, marked loose associations, impoverished thought content, marked illogical thinking, bizarre, disorganized, or catatonic behavior. Excluded uremia and drug causes.	0
3	Organic Brain Syndrome	Altered mental function with impaired orientation, memory or other intelligent function, with rapid onset fluctuating clinical features. Include clouding of consciousness with reduced capacity to focus, and inability to sustain attention to environment, plus at least 2 of the following: perceptual disturbance, incoherent speech, insomnia or daytime drowsiness, or increased or decreased psychomotor activity. Exclude metabolic, infectious or drug causes.	0
4	Visual Disturbance	Retinal changes of SLE. Include cytoid bodies, retinal hemorrhages, serious exudate or hemorrhages in the choroids, or optic neuritis. Exclude hypertension, infection, or drug causes.	0
5	Cranial Nerve Disorder	New onset of sensory or motor neuropathy involving cranial nerves.	0
6	Lupus Headache	Severe persistent headache: may be migrainous, but must be nonresponsive to narcotic analgesia.	0
7	Erebrovascular accident	New onset of cerebrovascular accident(s). Exclude arteriosclerosis	0
8	Vasculitis	Ulceration, gangrene, tender finger nodules, periungual, infarction, splinter hemorrhages, or biopsy or angiogram proof of vasculitis	0
9	Arthritis	More than 2 joints with pain and signs of inflammation (i.e., tenderness, swelling, or effusion).	0
10	Myositis	Proximal muscle aching/weakness, associated with elevated creatine phosphokinase/adolase or electromyogram changes or a biopsy showing myositis.	0
11	Urinary Casts	Heme granular or red blood cell casts.	4
12	Hematuria	>5 red blood cells/high power field. Exclude stone, infection or other cause.	4
13	Proteinuria	>0.5 gm/24 h. New onset or recent increase of more than 0.5 gm/24 h.	4
14	Pyuria	>5 white blood cells/high power field. Exclude infection.	4
15	New Rash	New onset or recurrence of inflammatory type rash.	0
16	Alopecia	New onset or recurrence of abnormal, patchy or diffuse loss of hair.	0
17	Mucosal Ulcers	New onset or recurrence of oral or nasal ulcerations.	0
18	Pleurisy	Pleuritic chest pain with pleural rub or effusion, or pleural thickening.	2
19	Pericarditis	Pericardial pain with at least 1 of the following: rub, effusion, or electrocardiogram confirmation.	0
20	Low Complement	Decrease in CH50, C3, or C4 below the lower limit of normal for testing laboratory.	2
21	Increased DNA Binding	>25% binding by Farr assay or above normal range for testing laboratory.	2
22	Fever	>38°C. Exclude infectious cause.	0
23	Thrombocytopenia	<1,00,000 platelets/mm3	1
24	Leukopenia	<3000 White blood cell/mm3. Exclude drug causes.	1
			24

SLE = systemic lupus erythematosus, SLEDAI = The SLE Disease Activity Index score.

### 3.2. Differential diagnosis of drug-induced lupus (DILE)

An exhaustive investigation was performed by a multidisciplinary team including clinical pharmacists to determine the underlying cause of SLE. The main differential diagnoses included the following: information on SLE was analyzed using the World Health Organization-Uppsala Monitoring Center criteria to evaluate whether they were connected to the drug (causality assessment).^[11]^

#### 3.2.1. Event with plausible time relationship to drug intake.

In our case, the patient presented with multiple systemic involvements that occurred during a long course of IFN-*α*2b treatment, which developed more severely after readministration.

#### 3.2.2. Cannot be explained by disease or other drugs.

No other anti-hepatitis B drugs, including nucleotide antiviral drugs, have been used since IFN therapy initiation. There was no family history of involvement of the immune system. Patients with a history of exposure to toxic substances were excluded from this study. No constitutional symptoms, such as weight loss, pronounced asthenia, or other clinical features suggestive of SLE, were present before IFN-*α*2b treatment. In contrast to idiopathic SLE, there is no female or racial predilection for DILE, and the average age of presentation is older than that of idiopathic SLE, as corroborated by our patient being a middle-aged male.^[12]^

According to previous literature, SLE onset and exacerbation might be associated with pathogen infection, notably viruses, alongside certain bacterial components that can also trigger activation of the immune system.^[13]^ Laboratory screening for suspected viruses, including cytomegalovirus, Epstein-Barr virus, human immunodeficiency virus, and hepatitis C, was negative. Qualitative and quantitative tests for Cryptococcus were normal, and the plasma 1-3-B-D glucan and Aspergillus galactomannan tests were negative. Specific mycobacterium and syphilis test results were negative, and the laboratory markers for Helicobacter pylori and parasitic infections were normal. Chest computed tomography did not reveal any features of infectious lesions. The main lupus-like manifestations associated with hepatitis B virus infection have been reported as glomerular diseases,^[14]^ including membranous nephropathy (MN), membranoproliferative glomerulonephritis (MPGN), immunoglobulin A nephropathy (IgAN), and focal segmental glomerulosclerosis (FSGS).^[15]^ The most common HBV-associated MN usually manifests clinically with varying grades of proteinuria and microscopic hematuria. Nephrotic syndrome, microscopic hematuria, and depressed serum complement levels are the most common presentations of MPGM.^[16]^ Although the diagnostic evaluation of proteinuria and hematuria is a common indication for renal biopsy, given the anemia and thrombocytopenia in our patient, biopsy was deferred. According to the Kidney Disease Improving Global Outcomes MN guidelines, a positive anti-PLA2R antibody test and nephrotic syndrome can confirm MN without a renal biopsy,^[10]^ while our patient’s anti-PLA2R antibody was negative, which was insufficient evidence for the diagnosis of MN. The presence of MPGN lesions implies that the pathogenic process has been present for some time and that other patterns of injury, such as endocapillary proliferative glomerulonephritis, mesangioproliferative glomerulonephritis, and crescentic glomerulonephritis, may occur as a result of the same process.^[10]^ Longitudinal renal diameter is a pivotal ultrasonic marker for chronic kidney lesion evaluation since it decreases as the glomerular filtration rate progressively declines. In chronic kidney disease, progressive renal mass loss, glomerular atrophy, and tubule-interstitial fibrosis are responsible for the decrease in renal blood flow, thereby reducing the amplitude of the spectral wave profile. The cortex may appear hyper- or hypoechoic with globular hypoechoic pyramids owing to interstitial edema.^[17]^ In this case, ultrasound did not detect abnormal signals or irregular kidney profiles, and abnormal longitudinal renal diameter and undefined differentiation between the cortex and medulla were not observed, which did not support the diagnosis of a chronic lesion caused by MPGN. Most commonly, IgAN is asymptomatic and follows a slowly progressive course, with approximately 25% to 30% of any cohort developing kidney failure within 20 to 25 years of presentation.^[18]^ Although a renal biopsy was not performed, the patient’s proteinuria, hematuria, and decreased creatinine clearance over a short period were consistent with acute kidney injury manifestations, and ultrasound features did not indicate chronic progression of IgAN lesions. Secondary FSGS, such as HBV-associated FSGS, is characterized by sub-nephropathy or renal-range proteinuria with normal serum albumin levels and low morbidity in adults unless infection persists. Two patients from a case report of HBV-associated FSGS were adolescents with HBV DNA copy numbers of 10^7^ IU/mL and 2 × 10^8^ IU/mL.^[19]^ Although this hypothesis was unlikely in our clinical case, an HBV DNA test was performed to evaluate the progression of the HBV infection and functional damage to the liver. No abnormal HBV proliferation was observed during IFN-*α*2b withdrawal, which makes this hypothesis less probable. Accordingly, there is no convincing evidence of infection-induced lupus erythematosus or glomerulonephritis.

#### 3.2.3. Response to withdrawal plausible.

Our patient showed no symptom improvement after drug withdrawal. However, the persistence of symptoms after drug withdrawal can also be explained by the severe adverse drug reactions.

#### 3.2.4. Event definitive pharmacologically.

The Food and Drug Administration Product Information reports that rare cases of autoimmune diseases including lupus erythematosus are observed in patients treated with IFN-*α*2b. In some clinical cases, IFN-induced ADR includes renal damage. LN is one of these, mainly presenting with proteinuria, abnormalities in urinalysis, and a transient increase in serum creatinine. The causal relationship between IFN and LN remains unclear, and the incidence of renal involvement during IFN therapy remains undetermined.^[20]^

#### 3.2.5. Rechallenge satisfactory, if necessary.

In this patient, a strong association was observed between IFN-*α*2b and serious adverse events. Therefore, IFN-*α*2b therapy was discontinued after hospitalization to prevent further physical damage. Based on previous findings, a multidisciplinary team diagnosed IFN-*α*2b as the cause of the SLE in this case.

### 3.3. Induction therapy and maintenance therapy

The kidney disease improving global outcomes guidelines recommend that LN treatment can be performed without renal biopsy confirming the diagnosis, and the standard protocol for active LN is high-dose glucocorticoids plus cyclophosphamide or mycophenolic acid (MPA).^[10]^ MPA pharmacokinetics varies considerably among patients, especially in the context of hypoalbuminemia and impaired kidney function,^[21,22]^ which was the case of our patient. To date, an adequate exposure dosage during initial treatment has not been fully established.^[23–25]^ For these reasons, clinical pharmacists considered cyclophosphamide a suitable option, which was accepted by physicians. The patient was administered of 40 mg methylprednisolone daily, 0.6 g cyclophosphamide monthly and 0.1 g hydroxychloroquine twice daily. The addition of trimethoprim-sulfamethoxazole after initiation of immunosuppressive therapy to prevent pneumocystis pneumonia is a serious complication in immunosuppressed patients. Against the glucocorticoid-induced osteoporosis, his scheme was added with 500 mg Calcium agent and 0.25*μ*g calcitriol daily during glucocorticoid therapy. The edema was treated with 30 mg of torasemide and 10 g of human serum albumin. IFN-*α*2b was discontinued after hospitalization, and 25 mg of tenofovir alafenamide fumarate daily was used to prevent HBV reactivation during immunosuppressive therapy. Pharmacists carefully explained the use of the medication and educated the patients about their precautions.

**Figure 4.**
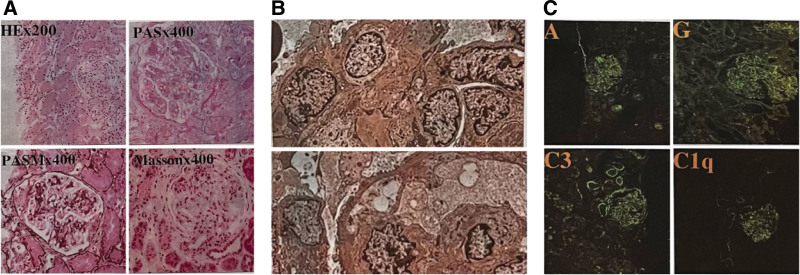
(A) Light microscopy reveals diffuse proliferative nephritis with track-like lesions, endo-capillary proliferation, and fibrocellular crescent in glomerular. The spike-like lesion structures on the lateral side of the glomerular basement membrane are observed. (B) Electron microscopy reveals diffuse and massive sub-endothelial dense deposits, intramembranous deposits and sub-epithelial deposits. (C) Immunofluorescence microscopy reveals staining on the glomerular capillary walls and partly on mesangial region with IgA, IgG, C3 complements and C1q. C1q = complement component 1q, IgG = Immunoglobulin G.

One month later, the patient was admitted to our department, with a subsequent course of cyclophosphamide. Contrary to the disappearance of leukopenia and pleural effusion (Fig. 1B), and improvement in anemia, thrombocytopenia, and decreased anti-dsDNA antibody levels, there was no improvement in proteinuria, hematuria, low complement, eyelid swelling, or leg pitting edema. Throughout the pharmaceutical ward rounds, clinical pharmacists noticed that intravenous 0.6 g cyclophosphamide was much lower than the dosage recommended in literature or guidelines.^[26]^ Clinical pharmacists have suggested that it is necessary to adjust the drug treatment strategies for these patients. The following adjustment plans were proposed after discussion: Patients should be given a dosage of 0.85 g cyclophosphamide based on a body surface area of 0.5 g/m^2^. If the patient’s condition did not improve, it could be increased to 1.71 g depending on the body surface area of 1 g/m^2^; If possible, cyclophosphamide may be replaced with agents with inhibitory effects on canonical or non-canonical type I IFN signaling pathways, such as baricitinib or sirolimus.^[27]^ The recommended dosage of baritinib is 2 mg daily, or the initial dosage of sirolimus is 2 mg per day, then the dosage was adjusted to maintenance according to a blood concentration range of 6 to 15 ng/mL.^[28,29]^ After thorough communication, the patient still preferred cyclophosphamide as treatment scheme due to its low price, and the clinician and clinical pharmacists finally decided to increase the dosage of cyclophosphamide from 0.6 g to 0.85 g. After the third course of cyclophosphamide, the platelet and hemoglobin levels gradually returned to normal; hence, a renal biopsy was performed. The biopsy findings were consistent with lupus nephritis class IV + V, as defined by the International Society of Nephrology/Renal Pathology Society classification (Fig. 4). At present, the patient has completed 6 courses of cyclophosphamide therapy, and no abnormal symptoms have been observed (Fig. 2C, 2D), except for a small amount of urinary protein (1.42 g/24 hours). HBV DNA test results did not reveal HBV progression.

Recurrence is an important factor that leads to aggravated organ damage and a poor prognosis. Therefore, an effective maintenance treatment is required. Calcineurin inhibitors (CNIs), sirolimus, MPA, and azathioprine are the available options for LN maintenance treatment. In terms of CNIs and sirolimus, drug concentration monitoring is required in patients treated with these drugs but not when azathioprine or MPA is used; this also has implications for compliance and accessibility. In addition, chronic CNIs nephrotoxicity should be considered. Among the preferred MPA or azathioprine, MPA is superior to azathioprine in maintaining renal response and preventing recurrence in patients with lupus nephritis who respond to induction therapy.^[30]^

Therefore, physicians and pharmacists determined that 500 mg MPA was an immunosuppressive regimen during the maintenance treatment phase.

## 4. Discussion

In this case report, we describe a male patient with HBV infection who developed SLE following long-term IFN-*α*2b therapy; the cause of SLE warrants further investigation. Conventional management or treatment is sufficient for idiopathic SLE. However, once DILE occurs, there is an urgent need to identify the drugs suspected to cause ADR. As part of a multidisciplinary team, clinical pharmacists first analyzed SLE and assessed whether it was related to IFN-*α*2b, using causality assessment criteria. IFN-*α*2b administration was discontinued to avoid further organ injury when it was identified as a trigger of DILE.

In previous reports, DILE generally showed only some non-serious symptoms (e.g., rash, arthritis, and serositis) and tended to resolve, requiring weeks to months after drug withdrawal.^[31]^ The main positive antibodies in DILE are ANA and anti-histone antibodies; anti-dsDNA is present only in rare cases and depends on the type of causative drug, for example TNF [tumor necrosis factor (TNF) -*α* inhibitors.^[32]^ Interestingly, our patient presented with severe organ involvement (e.g., LN and pancytopenia), which did not disappear after IFN-*α*2b discontinuation. Immunological tests revealed anti-dsDNA (1:581), ANA (1:1000), and anti-histone positivity, similar to idiopathic and TNF-*α* inhibitor-induced SLE. This case provides an opportunity to learn more about the modes of action that cause adverse reactions during long-term IFN-*α*2b therapy. The initial scheme was 0.6 g intravenous cyclophosphamide and 40 mg] according to the conventional regimen for idiopathic lupus. Pancytopenia, pleural effusion, and increased anti-dsDNA antibody levels were in remission with immunosuppressive therapy. However, proteinuria, hematuria, low complement levels, and edema did not improve. In this patient, the dosage and course of cyclophosphamide were less than the KIDGO guideline recommendation, which might be the cause of the poor response at the one-month follow-up. Accumulating evidence suggests that chronic or dysregulated activation of the type I interferon pathway contributes to SLE development.^[33]^ Type I interferons induce activation of canonical and non-canonical signaling, which might be the underlying cause of lupus in this patient Baricitinib or sirolimus were chosen based on their extrapolated efficacy for treatment of IFN-*α*2b-induced SLE due to inhibitory effects on JAK1/JAK2 kinase or monocyte-mTOR, respectively, which are important enzymes or cells in type I IFN-induced canonical or non-canonical signaling pathways.^[27]^ If observed, a prompt response to baricitinib or sirolimus supports this hypothesis. Unfortunately, the patient did not receive baricitinib or sirolimus regimens because of their high cost. Anifrolumab is also worth considering because of its unique mechanism of inhibition of the type I interferon pathway. However, its legitimate use has not been approved by the China Medical Products Administration.

HBV reactivation associated with immunosuppressive therapy is also a vital issue in this patient, as it is emerging as an important cause of morbidity in patients with previously well-controlled HBV infections. The niches of infected cells harbor either latent HBV DNA or low-level replicating HBV escape targeted by HBV-specific immune cells. Thus, these cells constitute a reservoir for persistent HBV infections. Although the size and nature of this reservoir in individuals with serological evidence of HBV recovery are unknown, it is clearly a source of HBV reactivation once immune control mechanisms are perturbed or suppressed.^[34]^ Chronic prednisone therapy, either medium-dose (10–20 mg orally daily) or high-dose (> 20 mg orally daily) for more than 4 weeks, increases the likelihood of HBV reactivation to a high risk of reactivation.^[35]^ Although there is no evidence that cyclophosphamide increases the risk of HBV reactivation due to its immunosuppressive potential, we expected that it would reactivate HBV. Therefore, anti-HBV prophylaxis should be considered until a definitive evidence is available. Before the patient received immunosuppressive therapy, pharmacists noticed this issue and proposed a preventive scheme (25 mg tenofovir alafenamide fumarate daily) that was accepted by the physicians.

To the best of our knowledge, most adverse drug reactions and high-risk medications are routinely identified by clinical pharmacists,^[36]^ and the choice of treatment for DILE, which entails personalized consideration of the balance between benefit and risk and is informed by data on short-term. Response and long-term efficacy and safety, potential adverse effects including infections and cumulative toxicities, quality of life, and factors relevant to patient experience and adherence, also benefit from pharmacists’ precise advice. When IFN-*α*2b is used for chronic hepatitis B, DILE should be considered, especially during long-term therapy, when the patient complains of anemia, bleeding, edema, and/or symptoms of renal injury.

## Acknowledgements

The authors thank the patients for allowing them to share their stories.

## Author contributions

**Investigation:** Hongxia Chen.

**Methodology:** Xiaoyan Qiu, Jingyi Wang.

**Supervision:** Xiaoyan Qiu.

**Writing – original draft:** Hongxia Chen.

**Writing – review & editing:** Hualing Wei.
